# Development and Validation of Quantitative Structure-Activity Relationship Models for Compounds Acting on Serotoninergic Receptors

**DOI:** 10.1100/2012/157950

**Published:** 2012-04-24

**Authors:** Grażyna Żydek, Elżbieta Brzezińska

**Affiliations:** Department of Analytical Chemistry, Faculty of Pharmacy, Medical University of Lodz, 1 Muszynski Street, 90-151 Lodz, Poland

## Abstract

A quantitative structure-activity relationship (QSAR) study has been made on 20 compounds with serotonin (5-HT) receptor affinity. Thin-layer chromatographic (TLC) data and physicochemical parameters were applied in this study. RP2 TLC 60F_254_ plates (silanized) impregnated with solutions of propionic acid, ethylbenzene, 4-ethylphenol, and propionamide (used as analogues of the key receptor amino acids) and their mixtures (denoted as S1–S7 biochromatographic models) were used in two developing phases as a model of drug-5-HT receptor interaction. The semiempirical method AM1 (HyperChem v. 7.0 program) and ACD/Labs v. 8.0 program were employed to calculate a set of physicochemical parameters for the investigated compounds. Correlation and multiple linear regression analysis were used to search for the best QSAR equations. The correlations obtained for the compounds studied represent their interactions with the proposed biochromatographic models. The good multivariate relationships (*R* ^2^ = 0.78–0.84) obtained by means of regression analysis can be used for predicting the quantitative effect of biological activity of different compounds with 5-HT receptor affinity. “Leave-one-out” (LOO) and “leave-*N*-out” (LNO) cross-validation methods were used to judge the predictive power of final regression equations.

## 1. Introduction

Serotonin (5-hydroxytryptamine, 5-HT) is a neurotransmitter in the central nervous system (CNS) that plays a significant role in migraine attacks, mood regulation, sleep, appetite, sexual function, anxiety treatment, depression, and schizophrenia. This neurotransmitter interacts with fourteen serotoninergic receptor subtypes, which are classified into seven families (5-HT_1–7_). Until now, we can explain the importance of functional groups 1–4; the physiological and therapeutic importance of the group 5–7 receptors is not yet known. With the exception of 5-HT_3_ receptor, which belongs to the family of ionotropic receptors, all are G protein-coupled receptors (metabotropic receptors) [1].

Many models of ligand binding describe interaction of ligands with serotonin receptors binding sites, but there are differences in them even for the same subtype of 5-HT receptor. Based on the available bibliographic data, it is established that the essential role in the creation of drug-serotonin receptor complex is played by the following amino acids: aspartic acid (Asp155), serine (Ser159), phenylalanine (Phe340), asparagine (Asn333), tryptophan (Trp200, 236, 367), tyrosine (Tyr370), and threonine (Thr196) [2–12]. A detailed description of the model binding sites was presented in earlier works [13, 14]. The information will make it possible to build an analytical model of interaction ligands with the 5-HT receptor and the initial prediction of potential biological activity of its ligands.

Chromatographic systems including chemical elements of the biological environment, simulating the conditions under which the studied compounds would act in a living organism (the so-called biochromatography) and thus the data obtained from such research (column, e.g., HPLC and thin-layer chromatography, TLC), are used very frequently in quantitative structure-activity relationship (QSAR) studies [15–24]. QSAR analysis consists in a mathematical treatment that is used to predict values of biological activity from physical characteristics of the structure of chemicals (molecular descriptors).

In the literature there are examples of biochromatographic data analyses, their implications for molecular pharmacology, and application in predicting pharmacological activity of drugs [25–32]. In such studies are employed both parameters obtained from the experimental interaction with the environment (e.g., biochromatography) and calculated physicochemical parameters, which result from the construction of chemical compounds.

This paper is a continuation of the studies [13, 14], whose purpose is to determine the possibility of using data obtained from thin-layer chromatography and computer-calculated physicochemical parameters to build regression equations that will predict the receptor binding affinity (p*K*_i_) and agonist (p*D*_2_) and antagonistic (p*A*_2_) activity of selected compounds acting on serotonin receptors. The choice of model compounds for the binding elements in the receptor structure was based on the literature data on the use of propionic acid to mimic aspartic acid structure in interaction with the ligands of the histamine H_3_ receptor [33, 34].

## 2. Experimental

### 2.1. Materials

Samples of the drugs **1–20** used in this work were purchased either as medical products or as standard substances [14]. All of them have biological activity toward serotoninergic receptors, see Table 1. The active substances were isolated from medical products with methods described according to specific monographs presented in the Polish Pharmacopoeia, and information is available in The Merck Index [35]. Pharmacological details are given elsewhere [14].

Propionic acid, ethylbenzene, 4-ethylphenol, propionamide, ethanol, isopropyl alcohol, and mobile-phase solvents were purchased from the Sigma-Aldrich (Steinheim, Germany).

### 2.2. Chromatography

Reversed-phase thin-layer chromatography system (RP2 TLC) was used for determination of the chromatographic data. The experiments were carried out twice in each variant of the stationary and mobile phase. TLC silica gel 60 RP2 *F*_254_ glass plates (silanized; 20 × 20 cm, Merck, Darmstadt, Germany) were used as the stationary phase. Chromatograms were developed in two mobile phases, denoted as *DS_A_* and *DS_B_*: (i) acetonitrile : methanol : buffer pH 7.4 (0.02 mol/L ammonium acetate) (40 : 40 : 20, v/v/v; *DS_A_*) and (ii) acetonitrile : methanol : methylene chloride : buffer pH 7.4 (60 : 10 : 10 : 20, v/v/v/v; *DS_B_*). All plates were first prerun for 1.5 h with the mobile phase, dried at ambient temperature, and then impregnated with 0.03 mol/L analogues of binding L-amino acid solutions ((a)–(g), *see below*) to obtain the corresponding biochromatographic models. The models were denoted as follows: (a) for propionic acid: S1, (b) ethylbenzene: S2, (c) 4-ethylphenol: S3, (d) propionamide: S4, (e) propionic acid + ethanol (1 : 1, v/v): S5, (f) propionic acid + ethanol + ethylbenzene (1 : 1 : 1, v/v/v): S6, (g) propionamide + isopropyl alcohol (1 : 1, v/v): S7.

The plates were impregnated with the solutions (a)–(g) by spraying in automatic TLC spray chamber (ChromaJet DS20, Desaga, Germany) and then air-dried. The impregnated and dried adsorbent layers were ready for chromatography. Additional plates (two for each type of mobile phase) were left clean for control analysis (C), without analogues of amino acids solutions.

The compounds **1–20** were weighed on analytical laboratory scales with 0.1 mg accuracy and then dissolved in methanol to obtain 1.0 mg/mL concentrations. The compounds in 1.0 *μ*L quantities were applied onto the previously prepared plates by means of Desaga AS30 TLC applicator (Desaga, Germany), at 1.0 cm intervals. The distance from the lateral edges was 2 cm. The start line was set at the level of 1.5 cm from the lower edge of the plate. The chromatograms were developed in a horizontal chromatographic chamber with an eluent dispenser, DS-II-20 × 20 (CHROMDES, Lublin, Poland) to the height of 12 cm above the lower edge of the plate. The duration of chromatograms development was 35 ± 2 min and 28 ± 2 min (for eluents *DS_A_* and *DS_B_*, resp.). The developed lanes were scanned densitometrically at 280 nm by means of a Desaga CD 60 densitometer with Windows-compatible ProQuant software (Desaga, Germany). For the particular compounds, the retention or retardation factor (*R*_f_)  values were read, and then the *R*_M_ values were calculated [36]: *R*_M_ = log⁡ (1/*R*_f_ − 1). The *R*_M_ values used for analysis constituted a mean from two reproducible experiments. *R*_M(S1)_– *R*_M(S7)_ and *R*_M(C)_ values for the analytes were presented in the course of the described quantitative analysis as S1–S7 and C, respectively, whereas the derivatives of these results were denoted with symbols: C-S(1–7) and S(1–7)/C. Using parameters of C-S(1–7) and S(1–7)/C has been justified in an earlier work [14]. The results of chromatographic analysis are presented in Table 2.

### 2.3. Calculation of the Molecular Descriptors

The semi-empirical method AM1 with Polak-Ribière algorithm (HyperChem v. 7.0 program) [37] and ACD/Labs v. 8.0 program [38] were employed to calculate a set of physicochemical parameters for the investigated compounds, see Table 3. The following set of variables was collected using (i) HyperChem program—the total energy (*E*_T_, kcal·mol^−1^), the binding energy (*E*_b_, kcal·mol^−1^), the heat of formation (Δ*H*_F_, kcal·mol^−1^), the total dipole moment (*μ*, D), the energy of the highest occupied molecular orbital (*ε*_HOMO_, eV), the energy of the lowest unoccupied molecular orbital (*ε*_LUMO_, eV), and the net atomic charge on the nitrogen atom (*Q*_*N*_), (ii) the module-QSAR Properties ChemPlus 2.1 included in Hyperchem software—the grid surface area (*A*_S_, Å^2^), the molar volume (*V*_m_, Å^3^), the hydration energy (*E*_H_, kcal·mol^−1^), the logarithm of the octanol/water partition coefficient (log *P*), the molar refractivity (*R*_m_, Å^3^), polarizability (*α*, Å^3^), and the molecular weight (*M*_W_, g·mol^−1^), and (iii) ACD/Labs 8.0 program—the distribution coefficient (log *D*), the polar surface area (PSA, Å^2^), the dissociation constant (p*K*_a_), the number of H-bond donors (HD), and the number of H-bond acceptors (HA).

### 2.4. Statistical Analysis

Stepwise multiple linear regression and correlation analysis were carried out using *STATISTICA* 9.0 program [39]. Values of biological activity (p*K*_i_, p*D*_2_ and p*A*_2_) of the analyzed compounds were used as dependent variables, as independent variables were applied in the chromatographic data and the calculated physicochemical descriptors.

The relationships between the behaviour of compounds **1–20** in chromatographic environments (C, S1–S7), their physicochemical properties, and their biological activity gave mathematical models whose statistical quality was estimated using of the following statistical indicators: the correlation coefficient (*R*), the squared correlation coefficient (the coefficient of determination, *R* ^2^), the variance ratio *F*, and the standard error of estimate (*s*), and the statistical significance (*P*-level) of the results was determined as *P* ≤ 0.05 (see Table 4).

The correlation between the biological activities with the various variables and the intercorrelation of descriptors was analyzed with the help of the correlation matrix. If two descriptors showed the correlation coefficient |*R*| > 0.5, one of them would be removed. The respective inter-correlation coefficients between the parameters occurring in the established regression models are given in Table 5. Evaluation of the best correlation models was carried out by validation of each model using general internal cross-validation procedures such as the “leave-one-out” (LOO) and “leave-*N*-out” (LNO). These kinds of internal validation are recommended if the number of compounds is small [40, 41]. The detailed procedures of these kinds of internal validation were described in an earlier work [14].

The cross-validated squared correlation coefficient (*Q* ^2^), predicted residual sum of squares (PRESS), standard deviation based on PRESS (*S*_PRESS_), and standard deviation of error of prediction (SDEP) were used to evaluate the predictive power the developed models. Some criteria for the reliability prediction and robustness of the models are suggested by authors [42–46]: *R* ^2^ > 0.6 and *Q* ^2^ > 0.5; *R* ^2^ ≥ *Q*_LO(N)O_^2^ and *Q*_LOO_^2^ ≈ *Q*_LNO_^2^.

## 3. Results and Discussion

The present work is a continuation of the previous studies [13, 14], whose purpose is to determine the possibility of using data obtained from thin-layer chromatography and computer-calculated physicochemical parameters to build regression equations that will predict the receptor binding affinity (p*K*_i_) and agonist (p*D*_2_) and antagonistic (p*A*_2_) activity of selected compounds acting on serotoninergic receptors. Similar studies have been carried out on compounds toward *β*-adrenergic [47, 48] and histamine [49–52] receptors.

In this study, we took advantage of the data based on the structure and function of serotoninergic receptors [12–22]. On the basis of the information, was established that the following amino acids: aspartic acid (Asp155), serine (Ser159), phenylalanine (Phe340), asparagine (Asn333), tyrosine (Tyr370), threonine (Thr196), and tryptophan (Trp200, 236, 367), located within 5-HT receptors play the most important role in ligands binding. This information made it possible to think out a hypothetical model of drug-serotonin receptor interaction, in which amino acids were introduced into the stationary phase of chromatographic environment [14].

The amino acids used to modify the stationary phase in previous work [14] contain amine and carboxyl groups which under biological conditions do not participate in the formation of the active complex. They remain within the protein structure forming peptide bonds. The presence of these active groups in a chromatographic system might lead to additional interactions with the compounds studied. In further experiments with our models of interactions with 5-HT receptors, we have thus used compounds (as analogues of the key receptor amino acids) with structures corresponding to those of crucial amino acids but without the amine and carboxyl groups which form peptide bonds in the receptor protein. And so for example, aspartic acid was therefore replaced with propionic acid, phenylalanine with ethylbenzene, tyrosine with 4-ethylphenol and asparagine with propionamide. Application of ethanol and isopropyl alcohol (as analogues of serine and threonine, resp.) as the substances modifying the stationary phase individually was devoid of sense. Ethanol and isopropyl alcohol were used only in multicomponent solutions for impregnation of plates. The choice of model compounds for the binding elements in the receptor structure was based on the literature data on the use of the propionic acid structure in studies of the interaction of aspartic acid with the ligands of the histamine H_3_ receptor [33, 34].

The correlation and the stepwise multiple linear regression analyses were carried out to answer the question whether there is any relationship between the behaviour of the compounds **1–20** in chromatographic environments S1–S7 and their biological activity (p*K*_i_, p*D*_2_, and p*A*_2_).

First, we analysed the relationship between the biological activity data and behaviour of the examined compounds in chromatographic environment of the control (C: without analogues of amino acids). The calculated correlation coefficient values (*R*) were (the regression equations are not presented in the text) 0.13 and 0.06 (p*K*_i_, *n* = 19, for *DS_A_* and *DS_B_* phases, resp.), 0.04 and 0.37 (p*D*_2_, *n* = 13, for *DS_A_* and *DS_B_* phases, resp.), and 0.02 and 0.10 (p*A*_2_, *n* = 16, for *DS_A_* and *DS_B_* phases, resp.). As indicated by the analysis, there was no correlation between serotoninergic activities of particular compounds **1–20** and their C-chromatographic data. It led to conclusions that the other significant relationships can depend upon the specific biochromatographic environment. Distinct relationships between values of biological activity and interactions data of the compounds **1–20** can be observed with all the models (S1–S7) (see Table 4).

Under the conditions of experiment with *DS_A_* mobile phase, distinct relationships were found for the compounds with acknowledged binding affinity p*K*_i_ to the 5-HT receptor ((1), Table 4) and determined agonistic activity p*D*_2_ ((6), Table 4). The binding affinity p*K*_i_ of the compounds to 5-HT receptor was described on the basis of models S1, S2, and S3. The relationship explains 68% of the variance and simultaneously describes the potential interactions between the ligands and amino acid residues: Asp155, Phe340, and Tyr370. In the case of agonistic activity p*D*_2_, chromatographic models S4 and S5 demonstrate a significant effect on regression equation, which explains 84% of the variance and simultaneously describes the potential interactions between the ligands and amino acid residues: Asn333, Asp155, and Ser159. All types of interactions between the structural elements of the receptor hydrophobic pocket and the chemical substance in the drug-receptor complex are represented in the above cases: ionic and hydrogen bonds, as well as stabilization of aromatic ligands rings by hydrophobic forces. For compounds with antagonistic activity p*A*_2_, the correlation was not statistically significant (11).

In the case of *DS_B_* mobile phase and for compounds with determined biological activity p*K*_i_ (2) and p*D*_2_ (7), the equations explain only 46–53% of total variance. The satisfactory results yielded the analysis of correlation between the data characterizing antagonistic activity p*A*_2_ and chromatographic parameters (*R* = 0.83; Equation (12)). The relationship explains 69% of the total variance and describes interactions of the ligands with amino acids: aspartic acid, tyrosine, serine, and phenylalanine (S1, S3, and S6 models).

At the next stage of the study, in the multiple regression analysis the molecular descriptors were employed as independent variables (Table 3). The contribution of the same descriptors in the development of regression equations was described in previous papers [13, 14]. The final mathematical models for biological activity (p*K*_i_ and p*A*_2_) explain 69% of the total variance ((3) and (13)), and model for p*D*_2_ explains only 57% of total variance (8).

Considering the role of molecular descriptors in prediction of biological activity, these parameters were included in the regression analysis together with chromatographic data. The share of the calculated molecular descriptors in the analysis of chromatographic models for *DS_A_* phase was presented in the form of (4), (9), and (14). The satisfactory result gave the regression analysis for compounds with p*K*_i_ and p*A*_2_ activity ((4) and (14)) where the mathematical models explain 60–68% of the total variance. For the dependent variable of p*D*_2_, the regression model explains 86% of the total variance, but the number of cases (*n* = 13) does not qualify for the introduction into analysis of the three independent variables.

Testing correlation for the development phase *DS_B_* yielded good results of regression analysis for biological activity p*K*_i_ and p*A*_2_ ((5) and (15)). In both cases, the mathematical models explain 80% of the total variance. For compounds with agonistic activity p*D*_2_, the correlation explains only 60% of the total variance ((17)-(18)). In the above models, you can see the contribution of both chromatographic data and the molecular descriptors.

It can be seen, in the case of these and previous studies [13, 14], that combining chromatographic data with physicochemical parameters has improved the results of QSAR analysis. In (3)–(5), (8)–(10), and (13)–(15), we can see clear influence of electronic (the net atomic charge on the nitrogen atom, the total dipole moment, the energy of the lowest unoccupied molecular orbital), thermodynamic (the distribution coefficient, the logarithm of the octanol/water partition coefficient, the molar volume, the heat of formation, and the molar refractivity), and structural (the number of H-bond donors) descriptors as independent variables determining biological activity.

As can be seen, *Q*_N_, log *P*, and *V*_m_ contribute positively and *μ*, log *D*, and HD contribute negatively to 5-HT receptor binding affinity. Antagonistic activity has positive influence on Δ*H*_F_, *R*_m_, and log *P* and negative influence on *μ*. Descriptor log⁡⁡*D* contributes positively to agonistic activity. Moreover, in all equations, One notes the influence of biochromatographic environments as the proposed models of drug-receptor interaction.

On the basis of such analyses, mathematical equations describing all the types of ligands interactions with 5-HT receptors can be proposed: (5), (6), and (15). The models, together with the statistical and validation parameters, are given in Table 5.

**Figure 1:**
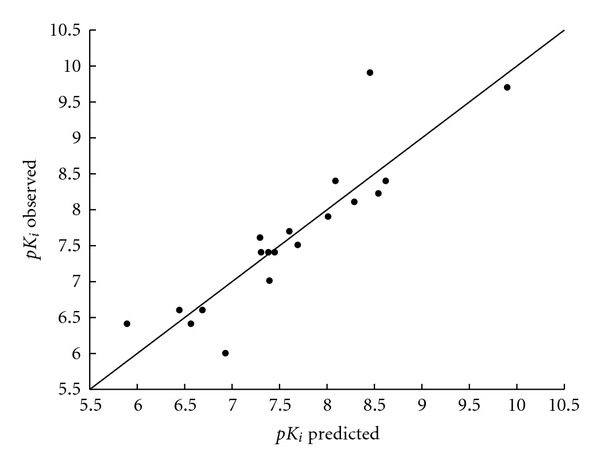
Observed versus predicted values of serotoninergic binding affinity p*K*_i_  according to (5).

**Figure 2:**
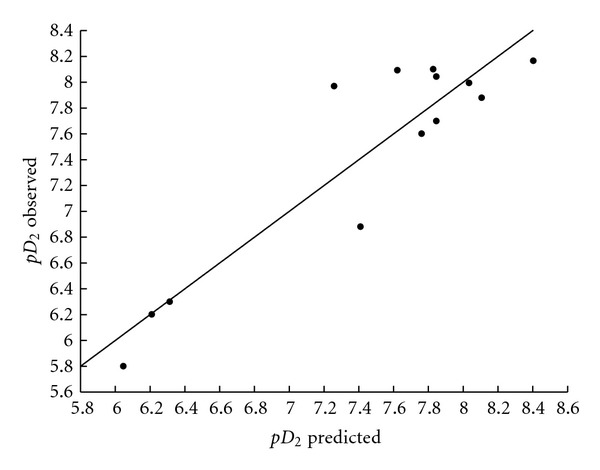
Observed versus predicted values of agonistic activity p*D*_2_ according to (6).

**Figure 3:**
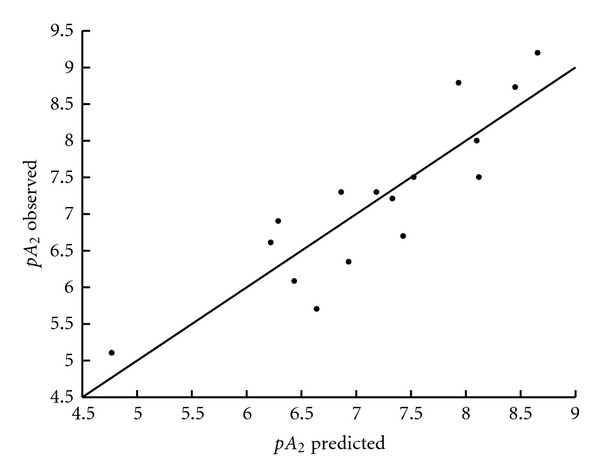
Observed versus predicted values of antagonistic activity p*A*_2_ according to (15).

Table 6 presents the correlation matrix, where it is shown that the selected descriptors from the above equations are not highly correlated.  Table 7 and Figures 1, 2, and 3 report the comparison of observed and predicted values of biological activity for (5), (6), and (15).

According to authors [45–49], terms for a reliable model: *Q* ^2^ > 0.5  and *R* ^2^ > 0.6, *Q*_LOO_^2^ ≤  *R* ^2^ ≥ *Q*_LNO_^2^ and *Q*_LOO_^2^ ≈ *Q*_LNO_^2^ are fulfilled in the equations in Table 5. The relation *R* ^2^_adj_ < *R* ^2^ confirms that models are not overparameterized.

These equations can be proposed as the tools for prediction of 5-HT activity of novel compounds characterized by various structures with 78–84% probability of obtaining a reliable result.

On the basis of the results described, it is clearly apparent that the simple analogues of amino acids important for ligand–receptor interaction are useful for building analytical models of the serotoninergic activity of drug candidates.

## 4. Conclusions

The QSAR models of compounds acting on serotoninergic receptors have been developed based on chromatographic data and electronic, thermodynamic, and structural descriptors. In all the types of chromatographic systems described above, models based on interaction of the compounds **1–20** with substances modifying the stationary phase have been found regression. The chemicals (propionic acid, ethylbenzene, 4-ethylphenol, and propionamide) used to impregnate the plates can represent the interaction of the compounds examined with the crucial amino acids (Asp, Phe, Tyr, and Asn, resp.). The proposed biochromatographic systems can describe an interaction which is possible between the ligands and the appropriate analogues of amino acids. A lack of correlation between the activity of the compounds and their behaviour in the control of chromatographic environment confirmed the important role of the presence of compounds modifying the stationary phase of chromatographic systems in construction of analytical drug-receptor interaction models. The predictive ability of models was demonstrated by using “leave-one-out” and “leave-*N*-out” cross-validation procedures. The results indicate that these models can be successfully used to predict the activity of 5-HT receptor ligands.

## Figures and Tables

**Table 1:** Known biological activity for compounds **1–20**.

No.	Compound	p*K*_i_^a^	p*D*_2_^b^	p*A*_2_^c^
1	Tiapride	—	7.97	5.10
2	Clopenthixol	7.60	7.99	7.30
3	Flupentixol	7.00	7.88	6.90
4	Trifluoperazine	7.90	8.04	5.70
5	Clozapine	6.00	—	7.50
6	Risperidone	9.90	8.16	6.69
7	Olanzapine	8.10	8.10	7.20
8	Tropisetron	8.40	—	6.60
9	Cyproheptadine	8.22	—	8.73
10	Trazodone	7.40	—	8.79
11	Mianserin	9.70	8.09	7.50
12	Pizotifen	7.40	—	9.20
13	Mirtazapine	8.40	6.88	7.99
14	Buspirone	7.70	7.70	6.35
15	Sumatriptan	6.60	5.80	—
16	Rizatriptan	6.40	6.30	—
17	Zolmitriptan	6.60	6.20	—
18	Cisapride	7.40	7.60	7.30
19	Serotonin	6.40	—	—
20	Propranolol	7.50	—	6.08

^a^p*K*_i_: 5-HT receptor binding affinity; ^b^p*D*_2_: agonistic activity; ^c^p*A*_2_: antagonistic activity.

**Table 2:** *R*_*M*_ values for the experiment with RP2 TLC system.

RP2 TLC *R*_M_ (*DS_A_* and *DS_B_*)								

Compound	*R*_M(C)_ (C)^a^	*R*_M(S1)_ (S1)^b^	*R*_M(S2)_ (S2)	*R*_M(S3)_ (S3)	*R*_M(S4)_ (S4)	*R*_M(S5)_ (S5)	*R*_M(S6)_ (S6)	*R*_M(S7)_ (S7)

Developing solvent *DS_A _*								
1	−0.140	−0.167	−0.131	−0.176	−0.185	−0.176	−0.105	−0.140
2	0.026	0.026	0.140	0.043	0.078	0.087	0.105	0.087
3	0.043	0.043	0.131	0.043	0.087	0.078	0.096	0.096
4	0.308	0.308	0.389	0.298	0.317	0.308	0.368	0.327
5	−0.026	−0.035	0.035	0.000	0.009	−0.009	0.035	0.009
6	−0.259	−0.269	−0.231	−0.317	−0.269	−0.368	−0.250	−0.240
7	0.078	0.087	0.176	0.078	0.096	0.096	0.149	0.140
8	0.122	0.105	0.149	0.061	0.061	0.000	0.131	0.105
9	0.269	0.259	0.288	0.222	0.240	0.231	0.278	0.259
10	−0.466	−0.466	−0.410	−0.410	−0.389	−0.421	−0.389	−0.368
11	−0.035	−0.035	0.000	−0.035	−0.026	−0.009	0.000	0.017
12	0.269	0.278	0.308	0.250	0.231	0.222	0.288	0.327
13	−0.122	−0.122	−0.061	−0.122	−0.122	−0.087	−0.070	−0.070
14	−0.337	−0.337	−0.231	−0.337	−0.317	−0.317	−0.269	−0.269
15	−0.078	−0.140	−0.070	−0.176	−0.222	−0.158	−0.122	−0.140
16	0.194	0.203	0.399	0.213	0.140	0.259	0.240	0.231
17	−0.078	−0.096	0.017	−0.114	−0.167	−0.070	−0.035	−0.105
18	−0.240	−0.231	−0.105	−0.213	−0.222	−0.213	−0.185	−0.185
19	0.087	0.087	0.194	0.035	0.017	−0.026	0.140	0.087
20	0.105	0.105	0.176	0.078	0.043	0.026	0.114	0.096

Developing solvent *DS_B _*								

1	0.009	0.009	0.035	−0.035	−0.026	0.026	0.026	0.009
2	0.269	0.259	0.259	0.213	0.222	0.278	0.250	0.222
3	0.250	0.259	0.240	0.194	0.213	0.278	0.231	0.185
4	0.513	0.489	0.477	0.454	0.466	0.501	0.466	0.432
5	0.194	0.194	0.149	0.122	0.140	0.203	0.149	0.105
6	0.070	0.052	0.043	0.000	0.035	0.061	0.026	−0.017
7	0.327	0.368	0.347	0.259	0.288	0.288	0.308	0.259
8	0.131	0.096	0.131	0.070	0.114	0.087	0.131	0.096
9	0.410	0.378	0.389	0.317	0.358	0.410	0.368	0.347
10	−0.250	−0.250	−0.308	−0.389	−0.317	−0.231	−0.288	−0.368
11	0.203	0.194	0.185	0.122	0.158	0.122	0.158	0.105
12	0.489	0.432	0.149	0.368	0.347	0.466	0.389	0.378
13	0.149	0.149	0.131	0.078	0.096	0.158	0.114	0.078
14	−0.009	0.026	−0.043	−0.140	−0.087	0.009	−0.017	−0.017
15	−0.009	−0.035	−0.017	−0.070	−0.070	−0.026	−0.026	−0.078
16	0.278	0.308	0.317	0.240	0.231	0.298	0.278	0.240
17	0.017	0.000	0.043	−0.035	−0.026	0.035	0.026	−0.026
18	0.122	0.140	0.105	0.035	0.061	0.131	0.096	0.061
19	0.203	0.222	0.231	0.149	0.140	0.176	0.194	0.149
20	0.203	0.222	0.185	0.114	0.131	0.176	0.203	0.158

^a^ *R*_M(C)_: retention parameter of the compounds in control environment of chromatography;  ^b^ *R*_M(S1−S7)_: retention parameters of the compounds in S1–S7 models environment of chromatography.

**Table 3:** Calculated molecular descriptors for compounds **1–20**.

Compound	*E*_T_	*E*_b_	Δ*H*_F_	*μ*	*ε*_HOMo_	*ε*_LUMO_	*A*_*S*_	*V*_m_	*E*_H_	log *P *	*R*_m_	**α**	*M*_*W*_	*Q*_N_	log *D *	p*K*_a_	PSA	HD	HA
1	−95473.52	−4458.85	−114.42	5.42	−9.22	−0.81	363.12	299.49	−5.38	−1.56	91.23	31.51	328.43	−0.28	−1.48	9.66	84.09	1	6
2	−102871.32	−5376.37	66.71	1.30	−7.68	−0.88	390.65	362.08	−7.20	−0.06	125.84	44.97	400.97	−0.26	5.06	3.40	52.01	1	3
3	−130832.64	−5768.48	−126.83	3.34	−8.19	−0.61	428.40	375.93	−7.16	0.73	126.33	44.61	434.52	−0.26	4.42	3.40	52.01	1	3
4	−121985.47	−5378.24	−77.03	3.37	−7.75	−0.54	397.66	353.23	−1.07	−0.19	119.66	41.84	407.50	−0.24	4.21	8.21	35.02	0	3
5	−86841.85	−4443.93	103.02	4.27	−8.12	−0.37	332.90	297.11	−2.85	−0.73	103.53	36.47	326.83	−0.26	3.28	7.14	30.87	1	4
6	−121479.92	−5930.86	−3.63	2.83	−8.88	−0.67	417.97	374.26	−3.95	0.63	118.48	43.54	410.49	−0.25	2.27	7.89	61.94	0	6
7	−80076.44	−4363.58	101.99	2.47	−8.20	−0.46	534.03	908.51	−2.75	−0.27	100.06	35.90	312.43	−0.26	2.68	6.08	59.11	1	4
8	−80841.49	−4306.45	−14.17	5.20	−8.83	−0.15	306.24	268.61	−4.73	−0.38	85.35	31.53	284.36	−0.22	1.07	10.00	45.33	1	4
9	−73412.66	−4717.17	78.67	0.93	−8.52	−0.23	320.59	290.43	−1.14	1.77	102.76	36.03	287.40	−0.24	4.86	8.95	3.24	0	1
10	−102872.02	−4942.24	104.46	3.65	−8.49	−0.53	386.68	336.51	−3.39	0.40	109.91	39.94	371.87	−0.25	1.58	6.73	42.39	0	6
11	−69008.06	−4266.06	78.00	1.69	−8.56	0.46	294.12	264.06	−0.67	0.94	91.21	32.26	264.37	−0.25	2.76	8.26	6.48	0	2
12	−72005.62	−4460.87	59.58	0.52	−8.66	0.06	315.58	285.55	−0.79	0.50	99.09	35.74	295.44	−0.26	4.49	9.04	31.48	0	1
13	−70511.88	−4151.08	82.98	1.40	−8.71	0.13	289.58	259.66	−1.56	1.16	87.08	31.55	265.36	−0.25	1.97	8.10	19.37	0	3
14	−110495.96	−5922.35	−34.38	4.11	−8.77	0.13	427.84	371.81	−1.85	1.18	109.35	42.11	385.51	−0.26	3.35	6.73	69.64	0	7
15	−81555.82	−4027.14	−16.02	2.30	−8.35	−0.50	327.29	270.64	−7.57	−1.53	85.92	29.34	295.40	−0.27	−1.38	9.49	73.58	2	5
16	−74102.91	−3978.93	139.36	5.47	−8.54	0.05	312.82	264.07	−6.38	−1.31	87.27	31.13	269.35	−0.27	−1.10	9.49	44.81	1	5
17	−82988.15	−4308.72	−22.22	6.51	−8.49	0.00	320.46	275.04	−6.13	−1.01	86.03	31.82	287.36	−0.28	−0.44	9.52	57.36	2	5
18	−140797.97	−6228.62	−162.08	3.89	−8.76	−0.11	480.40	412.47	−8.51	2.25	122.44	47.39	465.95	−0.27	2.60	7.77	86.05	3	7
19	−50258.52	−2618.46	1.22	3.41	−8.31	0.14	204.41	168.41	−15.76	−2.21	56.33	20.15	176.22	−0.35	−2.20	10.31	62.04	4	3
20	−73460.18	−4116.22	−55.72	2.21	−8.77	−0.51	311.49	260.89	−7.25	0.68	83.38	30.25	259.35	−0.30	1.37	9.14	41.49	2	3

**Table 4:** Regression models for the correlation between values of biological activity (*pK*_*i*_, *pD*_2_, *pA*_2_) and chromatographic data and molecular descriptors.

Equation no.	p*K*_i_	*R* ^ c^	*R* ^2d^	*F* ^ e^	*s* ^ f^	*p* ^ g^	*n* ^ h^
(1)^a^	a + bC-S2 − cS1/C + dS3/C − eS2/*C*	0.83	0.68	7.4815	0.6687	0.0019	19
(2)^b^	a + bC-S5 − cS5/C − dC-S4 + eC-S1	0.73	0.53	4.0085	0.8087	0.0226	19
(3)	a + b*Q*_N_ − c_*μ*_ − dlog⁡⁡*D*	0.83	0.69	11.0470	0.6387	0.0004	19
(4)^a^	a − bHD + c log *P* + dC-S5 + e*V*_m_	0.78	0.60	5.3183	0.7462	0.0081	19
(5)^b^	a − bHD + cC-S5 + dlog⁡⁡*P*−eC-S4	0.90	0.80	14.6095	0.5272	0.0001	19

p*D*_2_ =							

(6)^a^	a − bC-S4 + cC-S5	0.92	0.84	26.6280	0.3654	0.0001	13
(7)^b^	a − bS5/C − cC-S4	0.68	0.46	4.2261	0.6766	0.0467	13
(8)	a + b log *D* – *cε*_LUMO_	0.77	0.57	6.7003	0.6008	0.0142	13
(9)^a^	a − bC-S4 + cC-S5 – d*Q*_*N*_	0.93	0.86	19.2116	0.3560	0.0003	13
(10)^b^	a + blog *D* + cC − S4	0.77	0.60	7.4952	0.5814	0.0103	13

p*A*_2_ =							

(11)^a^	a − bC-S7 + cS1 + dS4/C	0.61	0.37	2.3231	1.0017	0.1267	16
(12)^b^	a − bC-S6 + cC-S3 + dS1/C	0.83	0.69	8.8524	0.7026	0.0022	16
(13)	a + b Δ*H*_F_− c**μ** + d*R*_m_	0.83	0.69	8.8893	0.7073	0.0022	16
(14)^a^	a − b*μ* − cS3/C − dC-S7	0.83	0.68	8.6594	0.7080	0.0025	16
(15)^b^	a + bC-S6 + cΔ*H*_F_ + *d*log⁡⁡*P*	0.88	0.78	14.0194	0.5934	0.0003	16

^a^Chromatographic parameters from the experiment in RP2 TLC *DS_A_* system. ^b^Chromatographic parameters from the experiment in RP2 TLC *DS_B_* system. ^c^The correlation coefficient. ^d^The coefficient of determination. ^e^The variance ratio *F*. ^f^The standard error of estimate. ^g^The significance level of the equation. ^h^The number of compounds used to derive the regression equation.

**Table 5:** QSAR statistics of significant equations.

Equation no.	Equation	*R*	*R* ^2^	*R*_adj_^2^	*F*	*s*	*p*	*Q*_LOO_^2^	SDEP	PRESS	*S*_PRESS_	*Q*_LNO_^2^	*n*
(5)	p*K*_i_ = 8.47 (±0.33)−12.96 (±4.99)HD + 19.98 (±4.47)C-S5+0.43 (±0.12)log *P * −0.34 (±0.12) C-S4	0.90	0.80	0.75	14.6095	0.5272	0.0001	0.70	0.5883	6.3052	0.5761	0.63	19

(6)	p*D*_2_ = 7.69 (±0.11)−15.89 (±2.18)C-S4 + 7.97(±2.36)C-S5	0.92	0.84	0.81	26.6280	0.3654	0.0001	0.73	0.4522	2.3390	0.4242	0.70	13

(15)	p*A*_2_ = 6.49 (±0.24) + 15.60 (±6.74) C-S6 + 0.01(±0.001) Δ*H*_F_+ 0.45 (±0.17) log *P *	0.88	0.78	0.72	14.0194	0.5934	0.0003	0.57	0.7633	8.6092	0.7335	0.55	16

*R* ^2^_adj_: the adjusted squared correlation coefficient; *Q*_LOO_^2^ and *Q*_LNO_^2^: the squared correlation coefficients of the LOO and LNO validation procedures, respectively; SDEP: the standard deviation of error of prediction; PRESS: the predicted residual sum of squares; *S*_PRESS_: the standard deviation based on PRESS.

**Table 6:** Correlation matrix of the biological activity (p*K*_i_, p*A*_2_ and p*D*_2_) and molecular descriptors used in (A) ((5) and (15)) and (B) (6).

	C-S4	C-S5	C-S6	Δ*H*_F_	log *P *	HD	p*A*_2_	p*K*_i_

(A)								
C-S4	1.00							
C-S5	−0.10	1.00						
C-S6	0.62	0.08	1.00					
Δ*H*_F_	0.10	0.22	0.34	1.00				
log *P *	0.21	−0.20	0.02	−0.35	1.00			
HD	−0.12	−0.07	−0.46	−0.54	0.16	1.00		
p*A*_2_	0.48	−0.10	0.62	0.60	0.23	−0.29	1.00	
p*K*_i_	−0.33	0.57	0.02	0.11	0.22	−0.39	−0.11	1.00

	C-S4	C-S5	p*D*_2_					

(B)								
C-S4	1.00							
C-S5	0.48	1.00						
p*D*_2_	−0.81	−0.03	1.00					

**Table 7:** Observed and predicted values of activity for Equations (5), (6), and (15).

Compound	p*K*_i_ (5)			p*D*_2_ (6)			p*A*_2_ (15)		
	Observed	Predicted	Residual	Observed	Predicted	Residual	Observed	Predicted	Residual
1	—	—	—	7.97	7.26	0.71	5.10	4.78	0.32
2	7.60	7.30	0.30	7.99	8.04	−0.05	7.30	7.19	0.11
3	7.00	7.39	−0.39	7.88	8.11	−0.23	6.90	6.29	0.61
4	7.90	8.02	−0.12	8.04	7.85	0.19	5.70	6.64	−0.94
5	6.00	6.93	−0.93	—	—	—	7.50	7.53	−0.03
6	9.90	8.46	1.44	8.16	8.41	−0.25	6.69	7.43	−0.74
7	8.10	8.29	−0.19	8.10	7.83	0.27	7.20	7.34	−0.14
8	8.40	8.62	−0.22	—	—	—	6.60	6.23	0.37
9	8.22	8.55	−0.33	—	—	—	8.73	8.45	0.28
10	7.40	7.39	0.01	—	—	—	8.79	7.94	0.85
11	9.70	9.90	−0.20	8.09	7.62	0.47	7.50	8.12	−0.62
12	7.40	7.31	0.09	—	—	—	9.20	8.66	0.54
13	8.40	8.10	0.30	6.88	7.41	−0.53	7.99	8.10	−0.11
14	7.70	7.61	0.09	7.70	7.85	−0.15	6.35	6.93	−0.58
15	6.60	6.69	−0.09	5.80	6.05	−0.25	—	—	—
16	6.40	6.57	−0.17	6.30	6.31	−0.01	—	—	—
17	6.60	6.45	0.15	6.20	6.21	−0.01	—	—	—
18	7.40	7.45	−0.05	7.60	7.77	−0.17	7.30	6.86	0.44
19	6.40	5.89	0.51	—	—	—	—	—	—
20	7.50	7.70	−0.20	—	—	—	6.08	6.43	−0.35
